# Developing a Novel Enamel Adhesive with Amorphous Calcium Phosphate and Silver Nanoparticles to Prevent Demineralization during Orthodontic Treatment

**DOI:** 10.3390/jfb14020077

**Published:** 2023-01-29

**Authors:** Ao Jia, Pei Wang, Fei Tong, Ziqiang Chen, Yunyun Deng, Haiyan Yao, Lianguo Wang, Yifan Liu, Hongshan Ge

**Affiliations:** 1 The Affiliated Stomatological Hospital of Nanchang University, Nanchang 330000, China; 2 The Key Laboratory of Oral Biomedicine, Nanchang 330000, China; 3 Jiangxi Province Clinical Research Center for Oral Diseases, Nanchang 330000, China

**Keywords:** orthodontic adhesive, antibacterial, amorphous calcium phosphate nanoparticles, dopamine, Ag nanoparticles

## Abstract

During fixed orthodontic treatment, white spot lesions are prevalent issues associated with cariogenic bacteria. This study aims to construct an orthodontic adhesive containing nanoparticles of amorphous calcium phosphate-polydopamine-Ag (NPA) fillers to combat white spot lesions. The NPA fillers were prepared and characterized by scanning electron microscopy (SEM), transmission electron microscopy (TEM), Fourier transform infrared spectroscopy (FTIR), and X-ray photoelectron spectroscopy (XPS). The biocompatibility of the fillers was evaluated. A colony counting test evaluated the antibacterial property of the fillers against *Streptococcus mutans (S. mutans)*. NPA fillers were mixed with orthodontic adhesive (Transbond XT) at different weight ratios (0, 0.1, 0.2, 0.3, and 0.5 wt.%). The shear bond strength and antibacterial properties were then further investigated. The results showed that NPA was prepared successfully, with good antibacterial properties. The cell survival rate of all groups of fillers was higher than 70%, showing good biocompatibility. Moreover, the shear bond strength of the orthodontic adhesive with 0.2 wt.% NPA fillers was 11.89 ± 1.27 MPa, meeting the minimal clinical bond strength requirements of 7.8 MPa. Furthermore, the orthodontic adhesive resin blocks and the extract displayed good antibacterial properties, with the number of colonies decreasing significantly (*p* < 0.001). Taken together, we think that an orthodontic adhesive with NPA may have a good application potential for the prevention and treatment of white spot lesions.

## 1. Introduction

White spot lesions are major complications following orthodontic treatment [1]. Due to the difficulties of removing the food residues surrounding the brackets during orthodontic treatment, cariogenic bacteria such as *Streptococcus mutans (S. mutans)* tend to adhere to the enamel surface around the brackets. Enamel demineralization occurs due to acid production, leading to white spot lesions [2]. The fluoride and chlorhexidine mouthwash are effective in preventing and treating white spot lesions, but their clinical application primarily depends on the patient’s compliance [3]. The addition of antibacterial components to orthodontic adhesives has been shown to effectively reduce bacterial adhesion around brackets, providing a new strategy for preventing and treating white spot lesions in orthodontic treatment.

The occurrence of white spot lesions is mainly due to the adhesion of cariogenic bacteria such as *S. mutans*, bacterial proliferation, acid production, local pH decreases of the enamel surface, and enamel demineralization. In contrast, both bacterial removal and enamel remineralization promotion can effectively prevent white spot lesions. Indeed, some researchers have added remineralization components such as boron nitride nanotubes (BNNTs) or mesoporous bioactive glass nanoparticles (MBN) to orthodontic adhesives and achieved good remineralization results [4,5]. Polymerizable quaternary ammonium monomers, which can covalently polymerize with the adhesive’s resin matrix components, have also been added to orthodontic adhesives. These have a broad antibacterial spectrum and can inhibit *S. mutans* on contact [4,5]. Thus, bacterial growth inhibition can be achieved while maintaining shear bond strength. Of all the polymerizable quaternary ammonium, dimethylaminohexadecyl methacrylate (DMAHDM) has the strongest antimicrobial properties [6]. However, such quaternary ammonium salts can only eliminate bacteria on the surface of the adhesive and cannot inhibit bacteria in the surrounding environment. Studies have previously attempted to evaluate the remineralization and mechanical properties of orthodontic adhesives containing calcium phosphate nanoparticles. The calcium phosphate remineralization system could release calcium and phosphorus ions and deposit them in the plaque and the surface of demineralized enamel, which can, in turn, elevate the local pH and facilitate enamel remineralization [7]. Nanoparticles of amorphous calcium phosphate (Nacp) are smaller than calcium phosphate in size and are precursors that release supersaturated calcium and phosphorus ions, acting as a reservoir of calcium and phosphorus ions. Nacp could rapidly neutralize acid to protect the tooth structure [8] and eventually convert to hydroxyapatite to remineralize the enamel effectively. Furthermore, it has been observed that Nacp is “intelligent,” as the release of calcium and phosphorus ions from Nacp increases significantly when the pH of the environment decreases, enhancing remineralization and effectively preventing enamel demineralization.

Silver has a broad-spectrum antibacterial effect. Ag nanoparticles have a smaller particle size and good biocompatibility compared to silver ions, with a long-lasting antibacterial ability and non-specific antibacterial properties [9,10]. Ag nanoparticles can inactivate important bacterial enzymes or interfere with bacterial DNA replication, leading to bacterial elimination. The application of Ag nanoparticles in polymethyl methacrylate, dental adhesives, and resin-modified glass ions can achieve sound antibacterial effects [11]. The homogeneous dispersion of Ag nanoparticles in the resin has an essential impact on the adhesives’ antimicrobial effect and mechanical properties. Some studies have used silver 2-ethylhexanoate as a precursor to form well-dispersed Ag nanoparticles in situ [12]. In contrast, silane coupling agent treatment and surface grafting modification for inorganic nanofilms are the two most common preventive strategies [13]. These surface modifications improve the dispersion and interfacial adhesion, but their practical applications are limited [14]. In recent years, the surface modification of nanofillers by mussel-induced dopamine (DA) has been a hot research topic for preparing organic–inorganic composites [15,16]. DA can self-polymerize and turn to polydopamine (PDA) on the surface of nanomaterials, which can enhance the dispersion of the added material by hydrogen bonding or Π-Π interactions with resin macromolecules, achieving a good affinity [17]. The catechol group in DA can reduce silver ions to Ag nanoparticles and firmly bind with them [18]. In addition, DA can effectively promote the remineralization of the enamel surface by increasing the volume of the hydroxyapatite crystals deposited on it, causing them to aggregate more densely and in a parallel pattern.

**Figure 1:**
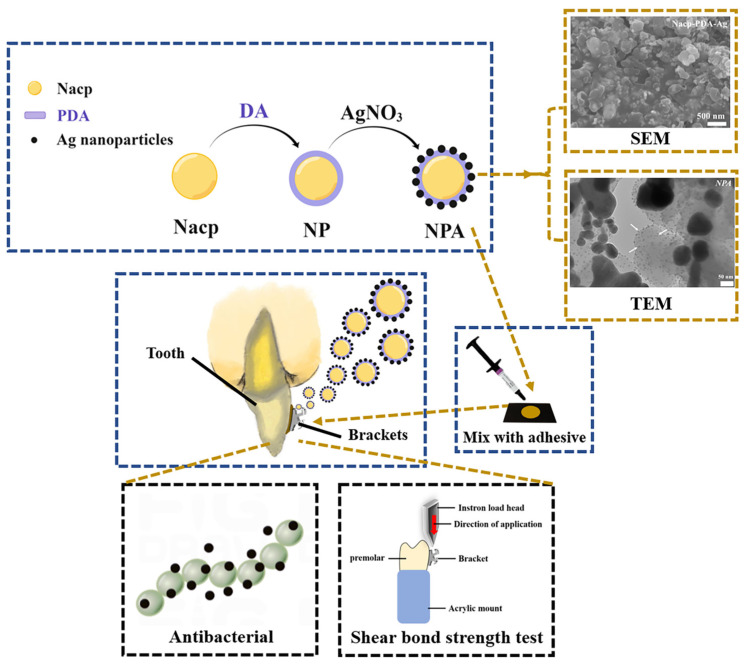
Schematic of the synthesis process of the NPA fillers and orthodontic adhesive containing NPA fillers with both antibacterial properties and a reasonable shear bond strength.

The present study uses a simple biological method to synthesize nanoparticles of amorphous calcium phosphate-polydopamine-Ag (NPA) fillers (Figure 1). The use of PDA as a medium for combining Nacp and Ag nanoparticles is the highlight of our article. Herein, the NPA fillers were added to the orthodontic adhesive in varying proportions, and the biocompatibility, antibacterial properties, shear bond strength, and adhesive residue index were tested to examine the property of the adhesives and provide a theoretical basis and new ideas for NPA applications. Collectively, we demonstrate that an orthodontic adhesive containing NPA is likely to be effective in preventing and treating white spot lesions.

## 2. Materials and Methods

### 2.1. Materials and Reagents

Nanoparticles of amorphous calcium phosphate were purchased from Nanjing Duly Biotech Co., Ltd. (Nanjing, China). Dopamine hydrochloride, silver nitrate, and anhydrous ethanol were obtained from Shanghai Aladdin Co., Ltd. (Shanghai, China). L929 was purchased from Ningbo Mingzhou Biological Technology Co., Ltd. (Ningbo, China). The acridine orange/ethidium bromide (AO/EB) double stain kit was procured from Beijing Runzekang Biological Technology Co., Ltd. (Beijing, China). 3-(4,5-Dimethyl-2-thiazolyl)-2,5-diphenyl-2H-tetrazolium bromide (MTT) was bought from Procell Life Science & Technology Co., Ltd. (Wuhan, China). *S. mutans* was purchased from China General Microbiological Culture Collection Center (Beijing, China). Brain Heart Infusion Broth (BHI) was obtained from Guangdong Huankai Biological Technology Co., Ltd. (Guangzhou, China). Orthodontic brackets were acquired from Hangzhou Sinya Co., Ltd. (Hangzhou, China). The orthodontic adhesive (Transbond XT) was purchased from 3M Co., Ltd. (Shanghai, China).

### 2.2. Preparation of NPA Fillers

Two steps were involved in the preparation of NPA. A total of 50 mg of Nacp was mixed with 50 mL of Tris buffer solution for ultrasonic dispersion. Then, an aqueous solution of dopamine hydrochloride (2 mg/mL) was prepared. The solution was put on a magnetic mixer and stirred continuously for 12 h at room temperature to allow the dopamine to self-polymerize on the surface of Nacp in order to obtain the Nacp-PDA (NP). After centrifugation, the NP was washed alternately with ethanol and deionized water thrice and lyophilized. The NP was then ultrasonically dispersed into AgNO_3_ solution (50 mM) and stirred continuously at room temperature for 4 h to obtain NPA.

### 2.3. Characterization

The surface micromorphology of three nanoparticles (Nacp, NP, and NPA) was observed using scanning electron microscopy (SEM). The layered structure of individual nanoparticles was investigated by transmission electron microscopy (TEM). Changes in the chemical composition of nanoparticle surfaces were observed using Fourier transform infrared spectroscopy (FTIR). X-ray diffraction (XRD) was used to analyze the state of nanoparticles. X-ray photoelectron spectroscopy (XPS) was conducted under ultra-high vacuum conditions to analyze the composition of three materials using an X-ray photoelectron spectrometer with an Al Kα-ray excitation source (hv = 1486.6 eV), and the high-resolution spectra of all samples were calibrated with the C_1s_ peak (284.8 eV) as the standard binding energy after the tests were completed. After mixing with NPA fillers, the adhesive surface roughness was then observed by SEM.

### 2.4. Preparation of the Antimicrobial Orthodontic Adhesive

NPA was added to the commercial orthodontic adhesive (Transbond XT) at different weight ratios (0, 0.1, 0.2, 0.3, and 0.5 wt.%), and the NPA was evenly mixed with Transbond XT on a glass plate using a mixing knife for 5 min. Air bubbles were removed. All adhesives were prepared within 30 min before use. The unused adhesive was discarded after the experiment.

### 2.5. Shear Bond Strength

#### 2.5.1. Shear Bond Strength

Fifty healthy premolars, with an average age of 20, extracted for orthodontic treatment were used for the shear bond strength test. Teeth were collected within 3 months, showing no cracks or caries. This study was approved by the Medical Ethics Committee of Stomatological Hospital Affiliated with Nanchang University; the approval code is 2022–029. The surface of the enamel was cleaned, and the roots were embedded in rectangular-shaped molds using self-consolidating resin. The samples were stored in deionized water and refrigerated at 4 °C. Using the random assignment method, the bonded specimens were divided into five groups, with 10 teeth in each group. The suggested bonding area of the enamel surface was acid-etched for 30 s. Various orthodontic adhesives were applied evenly on the orthodontic bracket’s base plate. A force gauge was used to apply a constant force to hold the bracket in position. An LED light curing lamp (light output intensity of 1200 mW/cm^2^) was used to irradiate for 20 s at a distance of about 1 mm from the bracket. The bonded specimens were immediately immersed in deionized water and stored in a constant temperature water bath at 37 °C for 24 h.

The specimens were fixed in the fixture of the universal testing machine, and the loading head was aligned parallel to the bonding surface. The shear bond strength test was performed from top to bottom with a loading speed of 0.5 mm/min until the bracket was dislodged. The maximum load (N) was recorded, and the shear bond strength of each group was calculated according to the bottom area of the orthodontic bracket (10.29 mm^2^) with the following formula:

Shear bond strength (MPa) = Maximum load (N)/Bracket bottom area (mm^2^)

#### 2.5.2. Adhesive Remnant Index (ARI) Scoring

The bonding area of the enamel surface after bracket removal was observed using a stereomicroscope, and the ARI was evaluated and recorded according to the following scoring criteria:

0 = No adhesive remnant on the enamel surface;

1 = Adhesive remnant on less than half of the bonding surface;

2 = Adhesive remnant on more than half of the bonding surface;

3 = Adhesive remnant on the entire bonding surface.

### 2.6. Analyses of Antibacterial Activity

#### 2.6.1. Preparation of Bacterial Suspensions and Strain Cultivation

*S. mutans* was thawed and incubated anaerobically in a 37 °C thermostat for 48 h. The colonies were proven to be pure by observing the morphology and staining the smear. The bacterial suspension was prepared with a 1 × 10^8^ CFU/mL concentration and incubated anaerobically in a BHI medium for 48 h.

#### 2.6.2. Antibacterial Tests of NPA Nanofillers

The NPA nanofillers were dispersed in the bacterial suspension (10^8^ CFU/mL) at concentrations of 25 μg/mL, 50 μg/mL, and 100 μg/mL, respectively. Under anaerobic conditions, the suspension was cultured for 6 h at 37 °C in a constant temperature incubator. Next, the suspension was diluted with PBS buffer, and 10 μL from each sample was aspirated and dropped onto the medium. Each experiment was repeated three times and incubated at 37 °C under anaerobic conditions for 24 h. The bacterial growth results were observed, and colonies of bacteria were counted and analyzed.

#### 2.6.3. Antibacterial Tests of the Adhesive

Transbond XT containing 0.2 wt.% NPA nanofillers and Transbond XT were placed in a cylindrical metal mold with a diameter of 6 mm and a height of 1 mm. The LED light curing lamp was used to irradiate the top and bottom of the resin at a distance of 1 mm for 20 s each (1200 mW/cm^2^) to obtain the resin blocks of Transbond XT+NPA and Transbond XT. The resin blocks (*n* = 3) were placed in 24-well plates, and 1 mL of the bacterial suspension was added to each well to completely cover the resin blocks. The specimens were incubated for 6 h under anaerobic conditions at 37 °C.

The resin blocks were removed, rinsed twice with PBS buffer, and then placed in a centrifuge tube with 1 mL of PBS buffer. The centrifuge tubes were ultrasonicated for 1 min to disperse the *S. mutans* adhering to the surface of the resin blocks in the PBS buffer. The remaining liquid was diluted, and 10 μL of each sample was aspirated and dropped onto the medium. Each experiment was repeated three times independently. The plates were placed upside down and incubated at 37 °C under anaerobic conditions for 12 h. The bacterial growth results were observed, and the number of colonies was quantified.

#### 2.6.4. Antibacterial Tests of the Adhesive Extract Solution

The resin blocks of Transbond XT+NPA and Transbond XT (*n* = 3) were placed in 24-well plates. A total of 1 mL of the bacterial suspension was added to each well to completely cover the resin blocks. The plates were incubated for 6 h under anaerobic conditions at 37 °C. The remaining bacterial suspension was diluted after the resin blocks were removed. A total of 10 μL of each group was aspirated and dropped onto the medium. Each experiment was repeated three times independently. The plates were placed upside down and incubated at 37 °C under anaerobic conditions for 12 h. In addition, bacterial colonies were counted.

### 2.7. Cytotoxicity Assay

#### 2.7.1. Live-Dead Cell Staining with AO/EB

Cells of L929 were used as experimental cells to evaluate the biocompatibility of NP nanoparticles. The L929 cells were incubated for 24 h at 5 % CO_2_, with a density of 8 × 10^4^ cells per well. The blank control group was added with 20% PBS buffer and the cell culture medium, and the experimental group was supplemented with 20 μL of a medium containing NP nanoparticles at different concentrations (10 μg/mL, 20 μg/mL, 50 μg/mL, 100 μg/mL). The cells were stained with AO/EB staining solution at 3 μL/well for 5 min at room temperature in the dark. The apoptosis of the cells in different groups was observed under an inverted fluorescent microscope.

#### 2.7.2. 1.3-(4,5-Dimethylthiazol-2-yl)-2,5-diphenyltetrazolium Bromide Assay (MTT)

NP was sterilized overnight under UV light and diluted to different concentrations (10 μg/mL, 20 μg/mL, 5 μg/mL, 100 μg/mL). An incubation time of 24 h was performed with L929 cells in a 96-well plate at a density of 8 × 10^4^ cells per well in 5% CO_2_. Then, the original culture medium was replaced with a cell culture medium containing different concentrations of NP nanoparticles. The blank control group received the cell culture medium only (*n* = 5). The cells were incubated with 5% CO_2_ at 37 °C for 24 h. Mitochondrial dehydrogenase in living cells can turn MTT into insoluble methanogenic particles, and the insoluble methanogenic particles can be dissolved in DMSO. A total of 20 μL of MTT solution (5 mg/mL) was added to each well, followed by a 4 h incubation period. The culture solution in the wells was then removed, and 150 μL DMSO was added. The crystalline methanogenic particles were fully dissolved by low-speed shaking for 10 min. The absorbance (OD) value was measured at 490 nm using an enzyme-linked immunoassay detector. The relative cell viability was calculated by setting the cell viability of the blank control group to 100% and by the formula below:

Relative cell viability = the mean OD value of the experimental group/the mean OD value of the blank control group.

### 2.8. Statistical Analysis

The Shapiro–Wilk test was used for the normal distribution. Levene’s test was used for the homogeneity of variance. The antibacterial properties were determined using a *t*-test. The statistical significance was set at *p* < 0.05. SPSS software (version 25.0, SPSS Inc., Chicago, IL, USA) was used for the statistical analysis. OriginPro8 was used for the mapping.

## 3. Results and Discussion

### 3.1. Characterization

**Figure 2:**
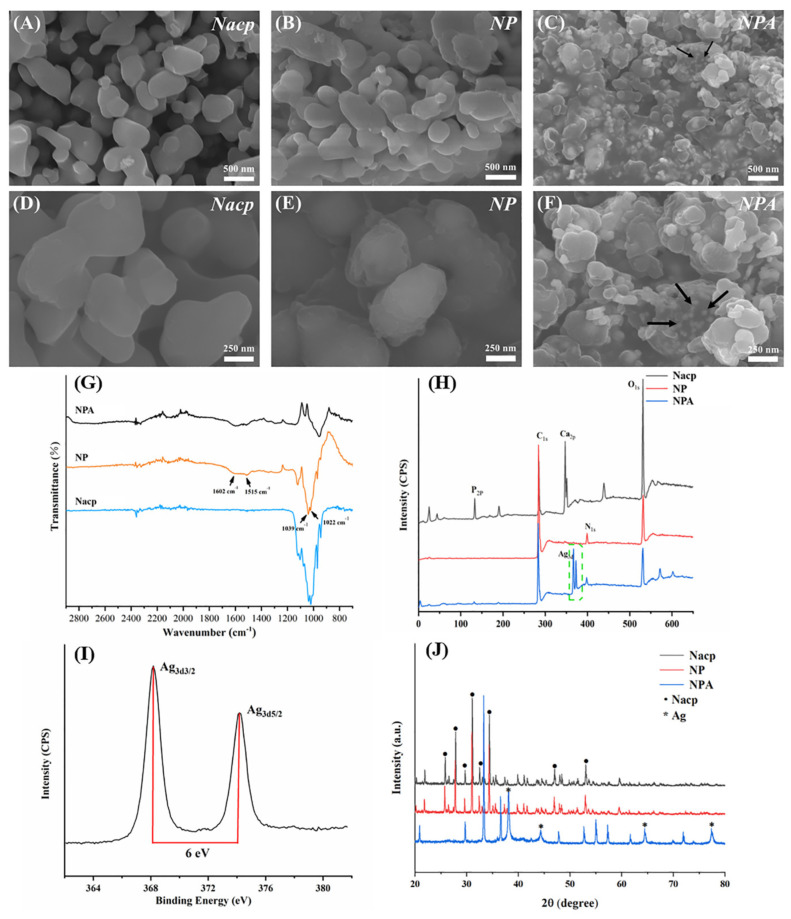
The characterization of Nacp, NP, and NPA with SEM (**A**−**F**), FTIR (**G**), XPS (**H**,**I**), and XRD (**J**). Figure (**I**) shows a magnification of the Ag signals in Figure (**H**) (the black arrows in Figure (**C**,**F**) indicate Ag nanoparticles).

As shown by SEM (Figure 2A–F), Nacp was spherical in shape, with a size of about 500 nm. In contrast to Nacp, DA self-polymerized and formed a polymer-like layer on the surface of NP and NPA. The characteristic signals of Nacp (C_1s_, O_1s_, P_2p_, and Ca_2p_) were confirmed by XPS analysis. NP and NPA showed a new N1s signal (400 EV) compared to Nacp in their XPS spectra, which is the characteristic signal of PDA (Figure 2H). The test results of XRD (Figure 2J) showed that the crystal structure of Nacp was similar to that of NP. PDA did not significantly change the crystal structure. FTIR analysis showed characteristic peaks of PDA in NP and NPA (Figure 2G). Notably, the peak at 1602 cm^−1^ corresponded to C = C and N-H in the aromatic ring, while the peak at 1515 cm^−1^ corresponded to the N-H vibrational peak in the amide group synonymous with the catechols and amines in PDA [19]. Our findings indicate that the polymer-like layer observed on the surface of NP and NPA under SEM is PDA.

**Figure 3:**
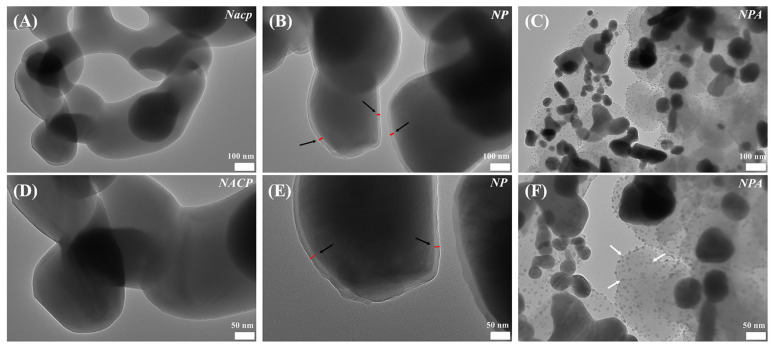
The characterization of Nacp, NP, and NPA using TEM (**A**–**F**). (The white arrows in Figure (**F**) indicate Ag nanoparticles.)

In addition, the characteristic peaks at 1022 cm^−1^ and 1039 cm^−1^ correspond to PO_4_^3−^ in Nacp. XPS and FTIR had a specific depth range for detecting the elemental composition of the material surface, while the layer of PDA wrapped around the surface of Nacp might affect the detection and analysis of the elemental composition in the depths of material. Therefore, the XPS and FTIR test results (Figure 2G,H) revealed that the number of calcium and phosphorus components in NP and NPA was smaller compared to the amount of Nacp. Silver, as a heavy element, could reflect electrons more strongly and present as bright spots under SEM. White spherical particles (30–50 nm) can be seen in NPA (Figure 2C,F), as shown by SEM, suggesting the presence of Ag nanoparticles (pointed to by black arrows in Figure 2F). The results of the TEM revealed the layered structure of individual particles. PDA was uniformly wrapped around the Nacp surface, forming a continuous layer (pointed to by the black arrows in Figure 3B,E). The white arrows in Figure 3C,F indicate the Ag nanoparticles. The diffraction peaks at 38.1°, 44.2°, 64.4°, and 77.3° are the characteristic peaks of silver (Figure 2J). XPS shows a double-peaked signal of Ag_3d_ in NPA at about 370 eV (Figure 2I), which is the characteristic signal of Ag^0^. After amplifying, the Ag_3d_ signal consists of two peaks at 368 eV and 374 eV (Figure 2I), with a spin-orbit spacing of 6.0 eV between the two peaks, which is due to the binding energy of Ag_3d3/2_ and Ag_3d5/2_ [19]. Together, the outcomes demonstrated that silver ions were successfully converted to silver atoms, and Ag nanoparticles were successfully reduced on the material’s surface. NPA fillers were prepared successfully with a simple method.

### 3.2. Antibacterial Test of NPA Nanoparticles

The rough surface of the orthodontic adhesive makes it easy for bacteria to adhere and colonize. The formed plaque is more difficult to remove. Ag nanoparticles can exert an excellent bacterial killing effect by releasing Ag^+^ [20]. Ag^+^ can interfere with the proteins on the bacterial cytoderm and change its structure and permeability, causing the contents to leak out of the bacteria [21,22,23,24]. In addition, Ag nanoparticles and Ag^+^ can inhibit the activity of enzymes in the bacterial respiratory chain, leading to bacterial death [25].

**Figure 4:**
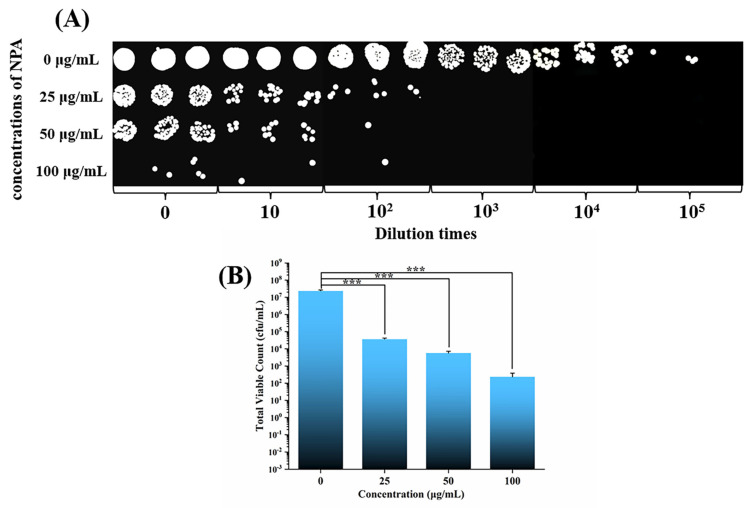
The outcomes of the NPA nanoparticles’ antibacterial test utilizing the film method. (**A**) Images of typical bacterial colonies of *S. mutans* following cocultivation with various concentrations of NPA nanoparticles. (**B**) Measurable outcomes connected with the bacterial quantities of *S. mutans* (*** represents *p* < 0.001).

Figure 4 demonstrates that the control group without NPA had a high number of bacterial colonies. The number of bacterial colonies decreased significantly after the addition of 25 μg/mL NPA compared with the control group (*p* < 0.001) (Figure 4B), demonstrating that the NPA fillers have good antibacterial properties at low concentrations [26]. No colonies were present when the bacterial suspension was diluted 10^3^ times. The number of colonies decreased further with the increase in the NPA concentration. The colony count results of all the groups with NPA fillers significantly differed from those of the control group (*p* < 0.001). The fillers concentration could affect the bonding of the bracket, so the specific number of NPA fillers to be added to the orthodontic adhesive should be analyzed and determined according to the shear bond strength.

### 3.3. Cytotoxicity

**Figure 5:**
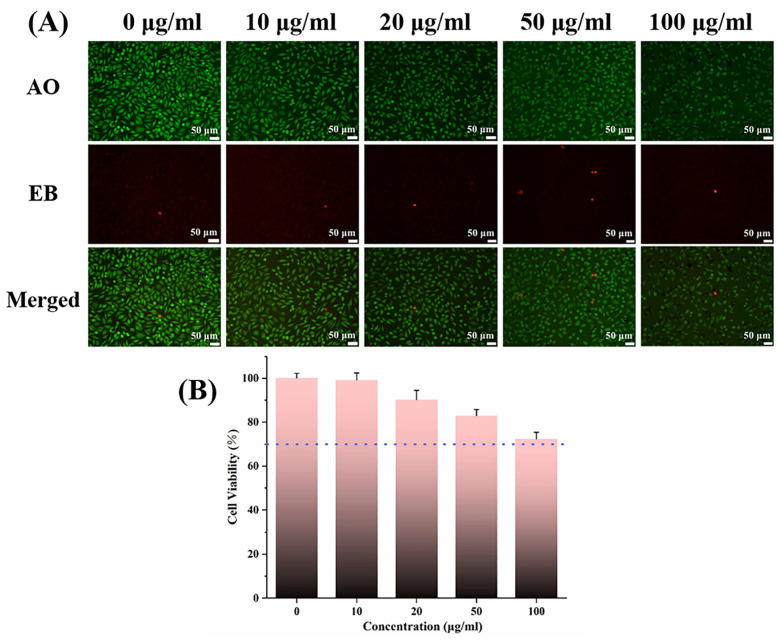
(**A**) AO/EB double staining after L929 cells were cultured with NP nanoparticles for 24 h. (**B**) The cytotoxicity of the NP nanoparticles determined using the MTT assay. The relative cell viability values that were all greater than 70% are depicted by the blue dashed line.

Cytotoxicity tests can effectively examine the biocompatibility of sample materials and lay the foundation for in vivo experiments. L929 cells have been used in this study for better sensitivity. Figure 5 shows the images obtained following AO/EB double staining after L929 cells were inoculated and co-cultured with different concentrations of NP nanoparticles for 24 h under a fluorescent microscope. Acridine orange (AO) can enter the nucleus of living cells and emit green fluorescence, while ethidium bromide (EB) can only enter the nucleus of apoptotic cells and emit red fluorescence. A large number of green cells with a normal morphology appeared in the images after five subgroups (0 μg/mL, 10 μg/mL, 20 μg/mL, 50 μg/mL, and 100 μg/mL) were stained with AO, while few red apoptotic cells were stained with EB. The blue dashed line represents a cell survival greater than 70% for all concentrations of NP (Figure 5B), meeting the International Standards Organization (ISO) requirements for material biocompatibility [27]. A small number of Ag nanoparticles are not toxic to fibroblasts and can maintain a good antibacterial effect [28]. Moreover, compounds with catechol structures such as PDA can reduce the toxicity of Ag nanoparticles. In addition, intraoral saliva flushing will reduce the concentration of Ag^+^ and toxicity, so the material may have fewer adverse effects on cells when applied to an oral environment with food, drink, and salivary stream washout.

### 3.4. Shear Bond Strength Test

**Figure 6:**
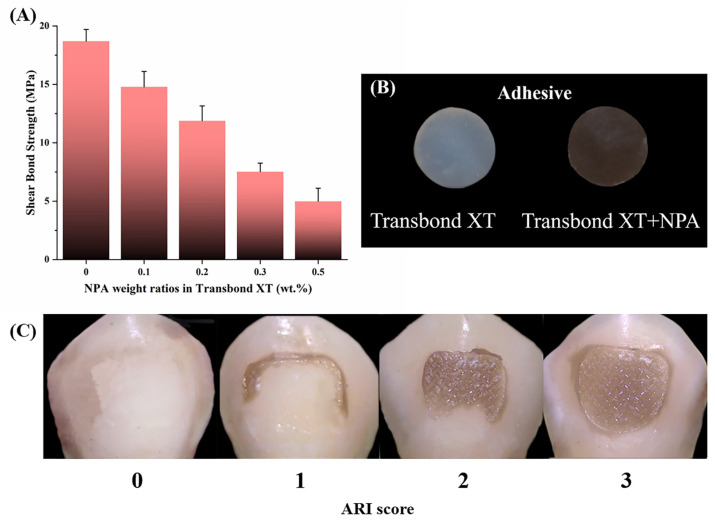
(**A**) Shear bond strength test results of the adhesive with varying proportions of NPA. (**B**) Resin blocks with or without NPA (Transbond XT and Transbond XT+NPA). (**C**) ARI scoring criteria.

**Figure 7:**
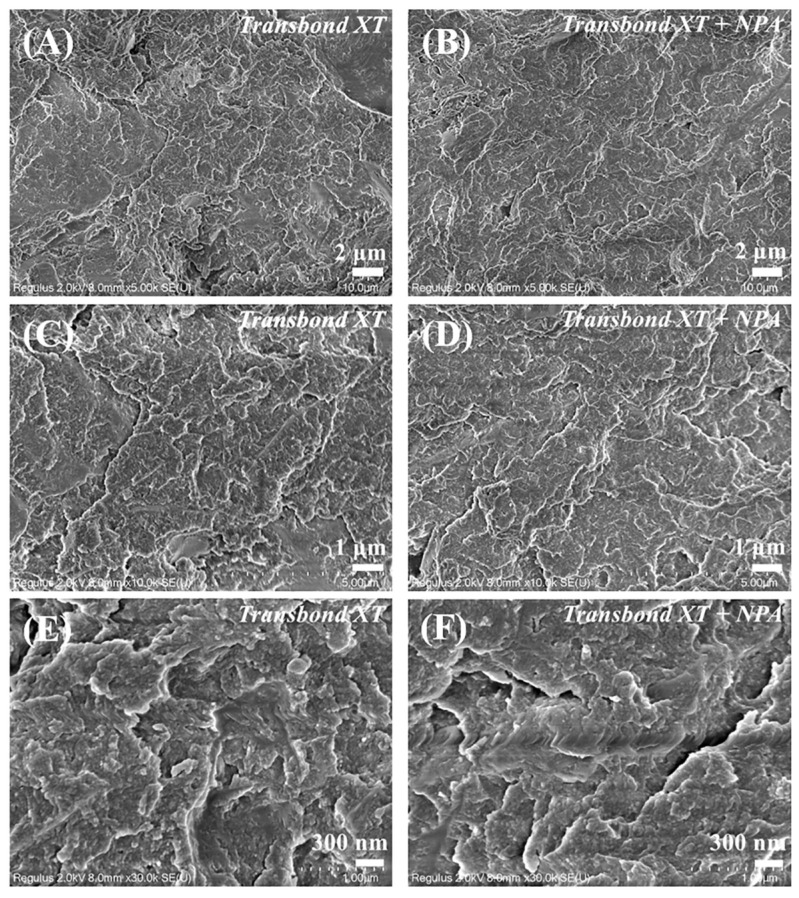
Images of the adhesive observed using SEM. Figure (**A**) corresponds to Transbond XT. Figure (**B**) corresponds to Transbond XT+NPA. (**C**,**E**) and (**D**,**F**) are the magnified views of (**A**,**B**).

To avoid bracket detachment due to insufficient bonding strength or enamel damage due to excessive bonding strength during bracket removal, the shear bond strength of an orthodontic adhesive should be appropriate. It was suggested that 7–9 MPa is favorable for providing adequate adhesion to the enamel. Increasing the value of the shear bond strength appropriately is conducive to preventing the bracket from coming off. The shear bond strength of commercially available orthodontic adhesives usually ranges from 10.1 MPa to 19.0 MPa [29], while the minimum clinically recommended bonding strength is 7.8 MPa [30]. As shown in Figure 6A, the shear bond strength was 18.70 ± 1.01 MPa in Transbond XT, decreasing gradually as the proportion of NPA incorporated increased. When the weight ratio of NPA fillers in Transbond XT was 0.3 wt%, the shear bond strength decreased to 7.53 ± 0.72 MPa, which is slightly below the minimum requirement. The shear bond strength of the adhesive with 0.2 wt.% NPA fillers was 11.89 ± 1.27 MPa, meeting the standard clinical requirements. Therefore, we use this group of adhesives in subsequent experiments. The surface of the adhesive was then observed by SEM (Figure 7). There was no significant difference in the surface roughness of the two kinds of adhesives before and after the addition of NPA fillers (Transbond XT and Transbond XT+NPA). No unevenly dispersed NPA fillers were observed on the surface of Transbond XT+NPA (Figure 7B,D,F).

In addition to the fillers content, the color of dopamine in the fillers may also be related to the decrease in the shear bond strength of the adhesive. PDA is black, and the fillers wrapped by PDA are also black. After adding the fillers, the orthodontic adhesive becomes darker, and the color of the adhesive gradually deepens as the fillers content increases, which may affect the curing light source reaching the deep layer of the resin and the polymerization of the orthodontic adhesive. The incomplete polymerization of the resin can affect the mechanical and physical properties of the adhesive, resulting in a decrease in bonding strength. The addition of NPA fillers to self-curing orthodontic adhesives can be considered in the future because self-curing adhesives can achieve the complete polymerization of resin by chemical curing without relying on curing light sources irradiation. In addition, the deepening of the adhesive color due to the increase in NPA fillers does not affect the curing of the chemically cured orthodontic adhesive, so the chemically cured orthodontic adhesive may allow for the addition of more fillers to achieve a better antimicrobial effect compared with that of the light-cured orthodontic adhesive. The correlation between the number of fillers added and the change in the shear bond strength of the chemically cured orthodontic adhesive should be further investigated.

**Figure 8:**
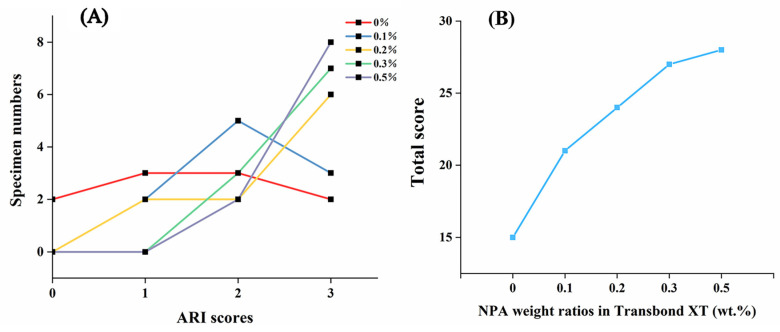
(**A**) Number of samples with different ARI scores for each group (*n* = 10). (**B**) Total ARI scores of different groups of adhesives.

The ARI scores reflect the adhesive residue on the enamel surface and can assist in detecting the shear bond strength. There are three types of adhesive fracture between the enamel and the bracket following bracket detachment. First, the adhesive fractures between the enamel and the resin. Second, the adhesive breaks inside the resin. Third, the adhesive breaks between the bracket and the resin. Although the residual adhesive on the tooth surface needs to be removed clinically for the second and third types of adhesive fracture, it is less likely to damage the enamel and is more in line with the concept of health in orthodontic treatment. The first type of adhesive fracture should be avoided whenever possible to minimize the damage to the enamel. The ARI scores in Figure 6A show that the scores of Transbond XT + 0 wt.% NPA were evenly distributed, while the number of bonded specimens with scores of 2 and 3 increased as the NPA content increased. Significantly, the total ARI scores and the adhesive residual on the enamel surface increased with the NPA fillers content increase (Figure 8B), indicating that the addition of fillers may affect the fracture type, with a shift from the first to the second and third types. Collectively, our findings reveal that the addition of fillers is beneficial in the protection of the enamel surface during bracket removal.

### 3.5. Antibacterial Test of the Adhesive

Next, the antibacterial ability of the materials on *S. mutans* was examined using both resin blocks and the resin block extract. The antimicrobial test of resin blocks can examine whether the NPA fillers kill the bacteria adhering to the resin surface. In comparison, the antimicrobial performance test of the resin block extract can examine whether the Ag nanoparticles in the fillers can effectively inhibit the growth of bacteria in the surrounding environment by releasing Ag^+^. The shear bond strength of Transbond XT + 0.3 wt.% NPA was too small to meet the clinical requirement, while that of Transbond XT + 0.2 wt.% NPA was enough (Figure 6A). Furthermore, 0.2 wt.% NPA was added in Transbond XT to prepare resin blocks to test the antimicrobial properties of the adhesive.

**Figure 9:**
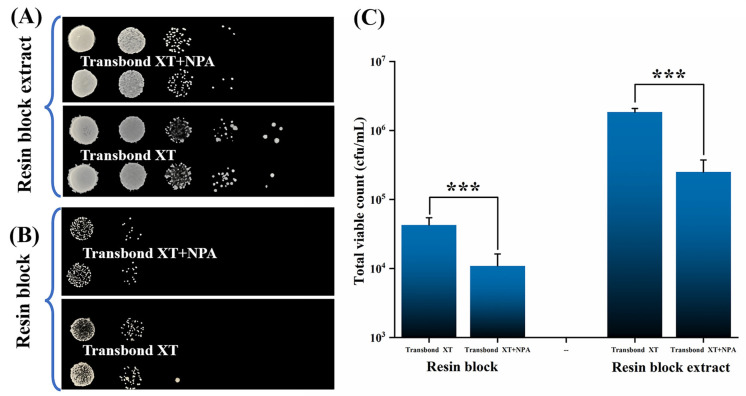
Antimicrobial properties test of adhesives using the colony counting method. (**A**) Images of *S. mutans* colonies adhering to the resin block surfaces. (**B**) Pictures of *S. mutans* colonies in the extract of the resin blocks. (**C**) Measurable outcomes of the *S. mutans* colony numbers (*** represents *p* < 0.001).

Figure 9B shows that the number of colonies on the resin blocks of Transbond XT+NPA was significantly less than that on the resin blocks of Transbond XT. Similarly, there was a significant difference following colony counting (*p* < 0.001) (Figure 9C). Ag^+^ released from the fillers was effective in killing the bacteria adhering to the surface of the resin blocks. The bacterial growth in the resin blocks extract of Transbond XT+NPA was significantly less than that in the group of Transbond XT (Figure 9A), which is consistent with the difference in the number of colonies (Figure 9C). Therefore, when the NPA fillers are added to the orthodontic adhesive, the Ag^+^ released from the Ag nanoparticles will kill not only the bacteria adhering to its surface but also the bacteria in the surrounding bacterial suspension [31]. The orthodontic adhesive containing 0.2 wt.% NPA fillers has good antimicrobial properties and can meet the clinical requirements of shear bond strength. There will be a good application prospect of the orthodontic adhesive with NPA in preventing and treating white spot lesions.

In this study, the antimicrobial properties and shear bond strength of an NPA-containing orthodontic adhesive were preliminarily determined through in vitro experiments. However, the oral environment is more complex and changeable. The physical properties of the adhesive could be affected by the polymerization, temperature, pH value, etc. Furthermore, Nacp in the adhesive can release calcium and phosphorus ions. PDA is rich in catecholamine groups, which are capable of binding to Ca^2+^ and enriching it on the enamel surface. The remineralization of the enamel surface could be promoted. White spot lesions could be prevented effectively. For further clinical applications, the adhesive’s physical properties and remineralization abilities will be studied in the future by simulating the intraoral environment with thermal and pH cycling.

## 4. Conclusions

Herein, the orthodontic adhesive with 0.2 wt.% NPA fillers demonstrated excellent antibacterial properties and met clinical shear bond strength requirements, indicating their great potential in preventing enamel white spot lesions following orthodontic treatment. Importantly, its preparation is simple, making it an excellent candidate for clinical applications. Nevertheless, further in-depth research is warranted to assess this experimental adhesive’s effectiveness. Its shear bond strength in the intraoral environment should be investigated.

## Data Availability

Not applicable.
